# Target trial emulation: teaching epidemiology and beyond

**DOI:** 10.1007/s10654-017-0293-4

**Published:** 2017-08-02

**Authors:** Jeremy A. Labrecque, Sonja A. Swanson

**Affiliations:** Department of Epidemiology, Erasmus Medical Center, P.O. Box 2040, 3000 CA Rotterdam, The Netherlands

Observational epidemiology is continually held to the standard of randomized trials. A typical epidemiology article references previous trials in the introduction (or reasons why trials are not feasible) and, when possible, compares the results to previous trials in the discussion. When the results from an observational study and trial disagree, we nearly always begin by questioning the former. Curiously, the methods section of an observational study—an undeniably crucial part of an article—rarely references trial methods or designs. Explicit target trial emulation aims to remedy this.

Target trial emulation is the application of design principles from randomized trials to the analysis of observational data, thereby explicitly tying the analysis to the trial it is emulating. The purpose is to improve the quality of observational epidemiology through the application of trial design principles, even when, or perhaps especially when, a comparator trial is not yet available or feasible [1]. In this issue of the *European Journal of Epidemiology*, García-Albéniz and colleagues [2] use observational data to compare effect estimates of colon cancer screening on cancer incidence over eight years of follow up using trial emulation alongside more commonly used analytic approaches. By precisely specifying their target trial’s eligibility criteria, treatment strategies, treatment assignment, follow-up period, outcome, causal contrast, and statistical analysis, they illustrate the value of emulating a well-specified target trial and show how common analytic approaches would be inappropriate.

Indeed, as others have also highlighted (e.g., [1–5]), emulating a target trial (and the formal causal frameworks it is compatible with) can help researchers identify and avoid unnecessary biases and provide a clear means for articulating the tradeoffs we may need to make in our observational studies. Unfortunately, we cannot always emulate the core elements of an ideal trial: it is usually not possible to emulate blinding, for example, or we may be forced to choose different eligibility criteria due to data availability considerations. Likewise, perhaps we cannot measure enough baseline confounders to emulate random assignment, and therefore consider alternative analytic strategies that may be valid but change the intervention or eligibility criteria in essential ways [6, 7]. Based on available measures or available knowledge, our intervention of interest may not be sufficiently well-defined, and describing the ill-defined target trial forces us to see that tradeoff [4, 8]. In some cases, even if we cannot emulate the ideal trial, we may still pursue the study while conscious of the compromises made. In other cases, we may realize the trial we are able to emulate is making tradeoffs that are too big and we may therefore choose to refine our questions or seek other data. Both of these outcomes are productive to the end-goal of improving epidemiologic research.

Here, we reflect on the contributions made by García-Albéniz et al. as well as other recent publications that embrace the trial analogy to discuss the broader potential impact of this idea. Given that others have previously discussed how trial emulation can improve epidemiologic research [1, 3, 4], we will focus on the impact trial emulation could have on the teaching of epidemiology as well as potential further methodologic advances it may inspire.

## Target trials in the classroom

Most important advances in epidemiologic methods in recent decades have been coupled with increased complexity both statistically and in the assumptions required for unbiased estimation. As much as g-methods, quasi-experimental methods, and other causal inference tools have improved the quality of observational research, understanding these methods well enough to apply them properly requires a substantial time investment. Trial emulation is different in this respect because the knowledge required to understand and apply it is already part of the traditional epidemiology curriculum. García-Albéniz et al. are not applying new methodologic principles per se: they are carefully applying design principles from trials to observational data where they have sometimes not been applied judiciously. In order to teach trial emulation to epidemiologists, introductory epidemiology courses require little augmentation. No new vocabulary or mathematical notation is needed beyond that already included in the teaching of trials. In fact, the inclusion of trial emulation creates a logical transition from the teaching of trials to observational research [9].

One nice consequence of teaching causal inference principles via trial emulation is that it clarifies immediately why some analytic practices are problematic. Consider how García-Albéniz et al. describe the two common but biased approaches they conduct alongside their preferred analysis. Without the trial emulation framework, the rationale behind these biased approaches can appear to be logical. However, if one were taught from the beginning to think in terms of trial emulation, their flaws could quickly be identified. For example, when examining the cumulative incidence curves of each analysis, it is clear that only the trial emulation analyses would be able to reproduce the crossing curves expected in a screening trial [10, 11]. In addition to avoiding biases, the framework also reveals opportunities for using available data more efficiently, although this involves more methodologic complexity that may be best taught in an intermediate or advanced course.

It seems clear that trial emulation should be a core component to how causal inference gets taught in epidemiology curriculum. The heuristic questions that remain are when and to whom it should be taught, how much emphasis should it be given, and whether potential negative consequences exist if trial emulation becomes commonplace. Educators need to weigh whether the trial emulation merits displacing other core material in epidemiology courses; alternatively, it is possible that its inclusion streamlines or better conveys the fundamentals of observational epidemiology that are already taught in introductory classes and better connects those fundamentals to the statistical analyses taught in advanced classes. Educators who teach epidemiology to non-epidemiologists (e.g., policy-makers; medical researchers from other disciplines) may also see their students’ comprehension improving, as trial emulation seems more readily accessible than teaching causal diagrams or counterfactual notation while still being able to convey the same foundational principles.

## Beyond the classroom

Beyond improving teaching and specific research applications, embracing a trial emulation framework can provide opportunities for epidemiologists to be more creative with the scientific process. (It will be important, of course, to formalize and assess any such broadened methods.) Here we briefly describe two such examples.

First, consider the setting from García-Albéniz et al. in which they emulate a target trial of screening colonoscopy knowing that actual randomized trials are on-going but will not be published for years to come. If their study and studies emulating related target trials in other data all demonstrate similar long-term benefits of colonoscopies, would we necessarily need to wait for a decade of follow-up time to accrue in the actual trials to gain confidence in the effects of colonoscopy? Even after a shorter follow-up time from the actual trials, it is possible to see whether the short-term causal effects estimated in observational studies closely mirror what is seen at that point in the trials. If they indeed map closely onto one another, it may serve as an indication that our observational study is on the right track and make us more confident that we can make colonoscopy recommendations much sooner than waiting for the full results of the trial. If they do not map closely onto one another, it may motivated us to re-examine the means by which we emulated random assignment (e.g., what unmeasured confounding could remain?). However, even if random assignment is successfully emulated, it is important to note that trial emulation will not guarantee comparability with real trials. Differences between populations being studied or other reasons the target trial protocol is not exactly aligned with the real trial may also cause estimates to differ [12]. By understanding when and why results agree, however, we can combine the powers of observational studies and actual on-going trials to support more timely conclusions, and continue to refine those conclusions as the trials continue.

Second, consider the rather common setting of studying some treatment’s effect on a rare outcome, such as the effect of statin medications on cardiovascular disease and death. To conduct a trial in a population of all indicated patients with a suitable sample size may not be feasible; instead, trialists may conduct a trial in a high-risk population (so that the outcome is not expected to be so rare) or they may study a surrogate outcome such as cholesterol levels. Neither is necessarily answering the questions most pressing to policy-makers, clinicians, or patients. With an observational study we could emulate the trial as conducted to see whether our estimates coincide with the trial. Armed with this “validation” of our observational study results, we can modify the target trial in our observational analysis to estimate other quantities such as effects in a more heterogeneous population or effects on endpoints of interest rather than surrogate endpoints. That is, we can combine results from actual randomized trials that did not target exactly the ideal question with the powers of observational studies to support more robust conclusions beyond those produced by a trial or an observational study alone.

## Conclusions

The degree to which explicit target trial emulation in observational studies aligns with results from well-conducted trials remains to be seen. The results thus far are encouraging (e.g., [2, 5, 13]) but studies that emulate a trial before the trial results are known, such as García-Albéniz et al. will be important. We believe, however, that the principles behind trial emulation ought to be adopted regardless because they encourage known good practices.

The immediate benefits of trial emulation have been outlined here and by others [1, 3, 4], but the impact of trial emulation goes beyond improving individual studies and into the classroom and how we can approach the scientific process as a whole. We thank García-Albéniz et al. for providing an excellent example to use in teaching trial emulation. When the colonoscopy randomized trials are published, we are sure their paper will be even more useful in the classroom, regardless of whether the results align. It remains to be seen, however, whether it will then serve to teach our future students about the value of trial emulation, the need for humility when estimating causal effects, the iterative nature of scientific findings, or most likely a combination of all of the above.
